# NEU1—A Unique Therapeutic Target for Alzheimer’s Disease

**DOI:** 10.3389/fphar.2022.902259

**Published:** 2022-06-29

**Authors:** Aiza Khan, Consolato M. Sergi

**Affiliations:** ^1^ Department of Laboratory Medicine and Pathology, University of Alberta, Edmonton, AB, Canada; ^2^ Division of Anatomic Pathology, Children’s Hospital of Eastern Ontario, University of Ottawa, Ottawa, ON, Canada

**Keywords:** NEU1, metabolic disease, sialidosis, neurodegeneration, Alzheimer’s disease, gene, gene therapy

## Abstract

Neuraminidase 1 (NEU1) is considered to be the most abundant and ubiquitous mammalian enzyme, with a broad tissue distribution. It plays a crucial role in a variety of cellular mechanisms. The deficiency of NEU1 has been implicated in various pathological manifestations of sialidosis and neurodegeneration. Thus, it is a novel therapeutic target for neurodegenerative changes in the Alzheimer’s brain. However, to manipulate NEU1 as a therapeutic target, it is imperative to understand that, although NEU1 is commonly known for its lysosomal catabolic function, it is also involved in other pathways. NEU1 is involved in immune response modulation, elastic fiber assembly modulation, insulin signaling, and cell proliferation. In recent years, our knowledge of NEU1 has continued to grow, yet, at the present moment, current data is still limited. In addition, the unique biochemical properties of NEU1 make it challenging to target it as an effective therapeutic option for sialidosis, which is a rare disease but has an enormous patient burden. However, the fact that NEU1 has been linked to the pathology of Alzheimer’s disease, which is rapidly growing worldwide, makes it more relevant to be studied and explored. In the present study, the authors have discussed various cellular mechanisms involving NEU1 and how they are relevant to sialidosis and Alzheimer’s disease.

## 1 Introduction

NEU1 belongs to the family of neuraminidases (sialidases). This enzyme mediates the removal of sialic acid (Sia) residues from glycoconjugates in vertebrates, subsequently regulating numerous physiological and pathological cellular activities (Smutova et al., 2014). Evidence suggests that NEU1 plays a role in various human disorders. These disorders include lysosomal disease, infectious disease, cancer, and neurodegenerative disorders, thus making it a critical therapeutic target (Glanz et al., 2019). The most associated condition with NEU1 is sialidosis, which occurs due to mutation in the neuraminidase gene (*NEU1*), located on 6p21.33 (Pshezhetsky et al., 1997; Uhl et al., 2002). Sialidosis is a lysosomal storage disease. Also, it is autosomal recessive, caused by a gene mutation (*NEU1*). The *NEU1* gene encodes the lysosomal sialidase NEU1 (Sergi et al., 1999). The subsequent deficiency of the enzyme activity causes a compromise in the process of degradation of sialoglycoproteins, consequently causing an accumulation of over-sialylated metabolites (Pshezhetsky et al., 1997). Sialidosis is a heterogeneous disorder with a diversified range of symptoms (Sergi et al., 1999; Sergi et al., 2001; Khan and Sergi, 2018; Sergi, 2020). Based on the onset and severity of clinical manifestations, sialidosis is divided into two types. Type I sialidosis is the less severe form, with a late onset of symptoms (d’Azzo et al., 2015). Typical symptoms of type I sialidosis, also known as cherry-red spot myoclonus syndrome, include progressive visual loss, bilateral cherry-red spots, and myoclonus, and are generally manifested during adolescence (Coppola et al., 2020). Type II sialidosis, in contrast, takes a more severe course and is further subdivided into three subtypes. In congenital type II sialidosis, patients are either stillborn or diagnosed at birth. The critical features include facial dysmorphism, skeletal dysplasia, mental retardation, and hepatomegaly and splenomegaly. The other two types are the infantile and juvenile types, in which sialidosis patients are born relatively healthy. However, soon after birth, these patients develop progressive visceromegaly and dysostosis multiplex. Also, there is moderate to severe mental retardation (Lowden and O’Brien, 1979). The most severe form is congenital sialidosis, which occurs entirely prenatally after the second trimester of pregnancy with non-immunological hydrops fetalis (NIHF) or isolated fetal ascites (Bonten et al., 2013).

**FIGURE 1:**
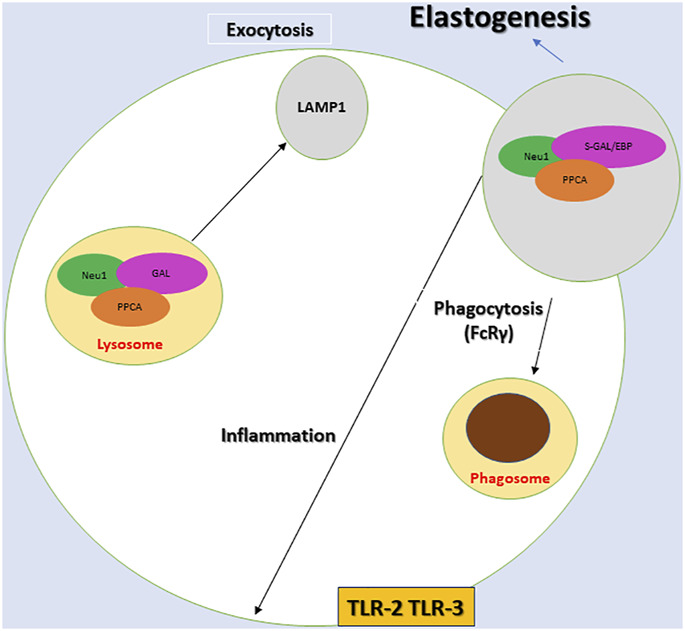
Diagram depicting the components of the multienzyme lysosomal complex, neuraminidase 1 (NEU1), protective protein/cathepsin A (PPCA) and β-galactosidase (Gal), and the associated proposed cellular mechanisms. NEU1 activation depends on PPCA within the NEU1-PPCA-Gal and NEU1-PPCA- S-Gal/EBP complex. The process of desialylation activates the receptors for phagocytosis (FcRγ), inflammation (TLR-2 and TLR3). NEU1 is also crucial for lysosomal exocytosis and elastogenesis. It should be noted that a variant of β-galactosidase S-Gal/EBP, NEU1, and CathA constitutes the elastin receptor that is targeted to the plasma membrane and is crucial in extracellular assembly of elastic fibers.

Currently, there is a lack of practical therapy for sialidosis due to the rarity of this disease (d’Azzo et al., 2015; Khan and Sergi, 2018). This lack of available therapeutic options has been attributed to the biochemical characteristics of NEU1. These characteristics are its tendency to aggregate, immune reactivity, and instability in the absence of PPCA (d’Azzo et al., 2015). Another critical aspect to consider is that although the catabolic role of NEU1 is profoundly crucial and remains central in the context of sialidosis. Nevertheless, recent studies suggest that NEU1 is involved in diverse cellular regulatory mechanisms. In addition to its role as a negative regulator of exocytosis (Pshezhetsky and Hinek, 2011), NEU1 plays a role in the modulation of the immune response (Pshezhetsky and Hinek, 2011), the generation of extracellular matrix, cell proliferation, and differentiation through desialylation of specific protein targets (Pshezhetsky and Hinek, 2011; Figure 1). It is essential to consider the diverse array of roles of NEU1, as it may lead to the use of NEU1 for other relatively common adult diseases. For example, numerous recent studies have demonstrated a therapeutic role of NEU1 in Alzheimer’s disease (AD), which is the most common type of neurodegenerative disease causing dementia [1213] (Serrano-Pozo et al., 2011; Chaudhary et al., 2018). Thus, as suggested before, if therapeutic targets such as NEU1 for rare diseases like sialidosis may prove helpful for other common conditions in adults, it may encourage the in-depth research and subsequent availability of therapeutic options for rare diseases and orphan diseases (d’Azzo et al., 2015).

In this study, the authors review the diverse roles of NEU1 in various cellular mechanisms, its potential substrates that have been reported in the literature so far, and its roles in different cellular mechanisms that have been associated with both sialidosis and AD. Also, the authors discuss the progress that has been made in the therapeutic interventions specific to sialidosis.

## 2 Cellular Mechanism and NEU1 Involvement

The NEU1 enzyme belongs to the family of sialidases. These enzymes remove sialic acid from the oligosaccharide chain (Yamaguchi et al., 2005; Miyagi and Yamaguchi, 2012). These sialidases, or neuraminidases, are widely distributed. They are required for many biological processes, including gangliosides and glycoproteins catabolism, plasma protein clearance, cell adhesion, and immunocyte function. NEU1 is the first and most abundantly found member of the sialidase family (Smutova et al., 2014). Substantially, NEU1 is a lysosomal exo-glycosidase. Functioning as a catalyst, it separates the terminal N-acetylated neuraminic acids (sialic acid) attached to the saccharide chains of glycoproteins, glycolipids, and oligo polysaccharides (Pshezhetsky and Ashmarina, 2001). One of the critical differentiating characteristics of NEU1 is that its active form exists only in a multienzyme complex along with other hydrolases: the glycosidase β-galactosidase (β-GAL) and the PPCA (Vinogradova et al., 1998). This association is essential for the stability and activity of all three enzymes, particularly NEU1, as it is critical for its catalytic activity (de Geest et al., 2002; Bonten et al., 2009). Here, it is essential to highlight that the PPCA acts as a chaperone/transport protein for NEU1. In fact, for many of its crucial activities, such as lysosomal compartmentalization, catalytic activation, and stability in lysosomes, NEU1 heavily depends on its association with PPCA (d’Azzo et al., 2015; Khan and Sergi, 2018). The interaction of NEU1 and PPCA takes place at an earlier biosynthetic stage. PPCA’s C-terminal portion is critical for its interaction with NEU1 and contains a binding site for NEU1 (de Geest et al., 2002; d’Azzo et al., 2015).

Interestingly, studies suggest that the binding domain in NEU1 for PPCA has an affinity for PPCA and NEU1. Hence, in the absence of PPCA, NEU1 tends to self-associate into chain-like oligomers. However, the binding of PPCA can reverse the self-association of NEU1 by causing the disassembly of NEU1-oligomers, with the inception of a PPCA-NEU1 heterodimeric complex (Bonten et al., 2009). Thus, it is plausible that the binding site of PPCA and NEU1 is critical. NEU1 mutations that affect its interaction with PPCA may cause disease, despite the active site of the enzyme remaining intact (de Geest et al., 2002). The absence of functional PPCA leads to another condition called Galactosialidosis (GS). It is a disease that occurs due to a combined deficiency of lysosomal neuraminidase and β-galactosidase. Patients with GS have clinical and biochemical features similar to those of sialidosis (de Geest et al., 2002). They are typically attributed to the absence of neuraminidase function (de Geest et al., 2002). Both sialidosis and GS are characterized by progressive vision impairment, bilateral macular cherry-red spots, skeletal and gait abnormalities, ataxia, seizures, and myoclonic syndrome. Knockout mouse models of sialidosis (NEU1-KO), as well as GS (CathA KO), lead to the development of systemic diseases closely resembling similar human conditions (Pshezhetsky and Ashmarina, 2013; d’Azzo et al., 2015; Khan and Sergi, 2018).

### 2.1 Various Mechanisms of Pathogenesis Attributed to NEU1

As noted above, In addition to the catabolic role of NEU1, recent animal studies have elucidated its role in diverse cellular regulatory mechanisms. The unique and essential role of NEU1 has been discovered in regulating exocytosis, modulation of the immune response, generation of cellular matric, and carcinogenesis through desialylation of specific protein targets (Pshezhetsky and Ashmarina, 2001; Pshezhetsky and Ashmarina, 2013; Smutova et al., 2014). In order to find a therapeutic role of NEU1 in various diseases, it is essential to examine the mechanisms of pathogenesis that have been attributed to NEU1, with the subsequent relevance in a more common condition. The following paragraphs are some of the notable roles of NEU1 discovered in the most recent studies.

## 3 NEU1 as a Negative Regulator of Lysosomal Exocytosis

The process of lysosomal exocytosis occurs under both physiological and pathological conditions (Pshezhetsky and Ashmarina, 2013). Also, this process takes place in a variety of numerous types of tissues. Thus, any alteration in this process ultimately results in an overall loss of tissue homeostasis (Andrejewski et al., 1999; Yogalingam et al., 2008; Pshezhetsky and Ashmarina, 2013). Initially, this calcium-dependent process was associated with secretory cells such as platelets, melanocytes, mast cells, neutrophils, and macrophages. However, there is evidence that lysosomal exocytosis occurs in almost all cell types, including neurons (Rodriguez et al., 1997; Kima et al., 2000).

Several studies have established that NEU1 is a negative regulator of lysosomal exocytosis (Yogalingam et al., 2008; Pshezhetsky and Hinek, 2011). It has been demonstrated that by affecting the levels of lysosomal associated membrane protein 1 (LAMP1), NEU1 negatively regulates lysosomal exocytosis (Yogalingam et al., 2008). LAMP1 has been known to be a crucial structural constituent of lysosomes. It is localized enormously in the limiting membrane of the organelle. It is scarce at the plasma membrane unless cells undergo exocytosis (Yogalingam et al., 2008; Pshezhetsky and Hinek, 2011), during which LAMP1 plays a critical role in the docking of lysosomes at the plasma membrane (Yogalingam et al., 2008). Studies have shown that NEU1-dependent cleavage of sialic acid by LAMP1 is vital in its intracellular trafficking. NEU1 controls the sialic acid content of the luminal domain of LAMP-1, its turnover rate, and subcellular distribution, which consequently determines the lysosomal exocytosis carried out by LAMP1. Animal studies have clearly shown that in *neu1* ^−/−^ bone marrow (BM)-derived macrophages, the lack of NEU1 results in hypersialylated LAMP-1, with a longer half-life and a tendency to accumulate in the plasmatic membrane. While silencing LAMP1 in NEU1-deficient cells leads to normalization of several lysosomes docked at the plasmatic membrane and a decline in the extent of lysosomal exocytosis (Rodriguez et al., 1997; Kima et al., 2000; d’Azzo et al., 2015).

### 3.1 NEU1’s Deficiency-Related Lysosomal Exocytosis and Pathological Manifestations of Sialidosis

It has been established that NEU1’s loss of function results in excessive extracellular release of lysosomal luminal contents from deficient cells of numerous tissues and organs (Rodriguez et al., 1997; Andrejewski et al., 1999; Kima et al., 2000; Yogalingam et al., 2008). Studies have elucidated the role of excessive lysosomal exocytosis in several pathological manifestations characteristic of sialidosis, such as splenomegaly (de Geest et al., 2002), extramedullary hematopoiesis (de Geest et al., 2002), muscle atrophy (d’Azzo et al., 2009), and hearing loss (Wu et al., 2010).

### 3.2 NEU1’s Deficiency-Related Lysosomal Exocytosis and Neuropsychiatric Changes

Importantly, this process has been implicated in pathologies related to the central nervous system, such as neurodegeneration (d’Azzo et al., 2015; Khan and Sergi, 2018) and changes in the emotional behavior of an animal model (Ikeda et al., 2021). It is intriguing that the role of NEU1 in AD may be supported by very recent studies performed on the Neu1-KO zebrafish model. Ikeda et al. suggested the link of Neu1 with altered emotional activity, pointing to a deregulated lysosomal exocytosis, probably as suggested 8 years earlier (Annunziata et al., 2013; Ikeda et al., 2021).

### 3.3 NEU1’s Deficiency-Related Lysosomal Exocytosis and Altered Emotional Activity

Studies investigating the effect of NEU1 deficiency on emotional activity remain limited. However, in one exciting study, behavioral analysis was performed on Neu1-knockout zebrafish (*Neu1*-KO) (Ikeda et al., 2021). It was noticed that although Neu1-KO zebrafish exhibited normal swimming patterns similar to wild-type (WT) zebrafish, there was a decline in shoals, as well as altered patterns of interaction with different fish species. Moreover, the aggression test also demonstrated a notable reduction in aggressive behavior in Neu1-KO zebrafish. Also, in Neu1-KO zebrafish, a downregulation of the anxiety-related genes of the hypothalamic-pituitary-adrenal axis was noticed. The authors reported the underlying mechanism to be the upregulation of *lamp1a*, an activator of lysosomal exocytosis, resulting in the accumulation of several sphingoglycolipids in the Neu1-KO Zebrafish brain. Interestingly, studies have also reported that induction of anxiety may cause an upregulation of *Neu1* in the zebrafish brain along with a simultaneous reduction in Lamp1 levels. It suggests that the significance of enhanced Neu1 and reduced lysosomal exocytosis might be crucial to suppressing boldness/exploratory activity under circumstances that require caution (Ikeda et al., 2021). Overall, it can be suggested that NEU1 deficiency may also lead to abnormal emotional behavior. It can be attributed to neuronal dysfunction induced by lysosomal exocytosis.

### 3.4 NEU1’s Deficiency-Related Lysosomal Exocytosis and Neurodegeneration and Links to Alzheimer’s Disease

Several studies have suggested a link between NEU1 and neurodegeneration (Grimm et al., 2012; Annunziata et al., 2013; Boutry et al., 2018; Khan and Sergi, 2018). In one study, *Neu1* ^−/−^ mice showed a brain phenotype with signs of early aging, with the presence of amyloid deposits similar to the plaque that is characteristic of AD, which is the most common cause of dementia among older adults (Javaid et al., 2021). The histopathological examination of the *Neu1* ^−/−^ brain revealed several multifocal, eosinophilic deposits of varying size and shape, particularly in the CA3 region of the hippocampus and the fimbria with enhanced lysosomal exocytosis (Annunziata et al., 2013). These deposits were noted to have proteinaceous material stained positive with the Congo red/Chrysamine-G derivative Methoxy-X04, a compound with a high affinity for amyloid. It is to be noted that studies performed on the zebrafish model have also reported similar results (Annunziata et al., 2013). Several observations in the study asserted that the NEU1 loss might contribute to the phenotypes of AD. For instance, it was noticed that lack of NEU1 was associated with over-sialyation of amyloid precursor protein APP, demonstrating APP to be a substrate for NEU1. Also, in *Neu1* ^−/−^ hippocampal neurons, the accumulation of amyloid precursor protein (APP) was noticed to take place at a very early age. Moreover, the levels of secreted Aβ42 appeared higher in *Neu1* ^−/−^ cerebrospinal fluid and in the medium of Neu1^−/−^ hippocampal cultures compared to the corresponding control samples. Finally, cerebral injection of NEU1 in an established AD mouse model showed a considerable amount of reduction in β-amyloid plaques (Annunziata et al., 2013). In addition, in a study carried out on Neu1-KO zebrafish, in the brain of Neu1-KO zebrafish, an abnormal accumulation of GM1 ganglioside was reported (Ikeda et al., 2021). This finding is crucial since it demonstrates another correlation between NEU1 and AD. Gangliosides (a family of sialic acids with glycosphingolipids) are known to be present in high concentrations in neuronal and glial membranes and are involved in the development and maintenance of neuronal cells and tissues (Yanagisawa, 2007; Grimm et al., 2012). The involvement of these gangliosides in AD pathology is a well-established fact. Various studies have demonstrated that the concentration and composition of gangliosides are different in the AD brain, both in patients and in animal models. These gangliosides appear to accumulate in the neuronal membranes and contribute to the formation of amyloid fibrils. Notably, GM1 ganglioside (GM1), has been shown to cause upregulation in AB production and is associated with early pathological changes of AD, playing a key role in Aβ assembly in the AD brain (Yanagisawa, 2007; Grimm et al., 2012). As mentioned earlier, in NEU1-deficient-brain, an abnormal amount of GM1 ganglioside was noted to be accumulated in the brain. Hence, it is plausible that another pathway in which NEU1 deficiency may lead to AD pathology is GM1 accumulation (Ikeda et al., 2021). However, the overall data regarding the storage of gangliosides in human sialidosis patients remains inconclusive. Yet some studies have elucidated the role of NEU1 in ganglioside degradation (Boutry et al., 2018). More research in this area may provide additional crucial details. In light of the abovementioned findings, it can be said that NEU1 loss of function may result in an AD-like phenotype in the sialidosis mice, thus establishing loss of NEU1 enzyme activity as a risk factor for the development of this disease (Annunziata et al., 2013; Bonten et al., 2014).

## 4 NEU1 and Immune System Modulation

NEU1 has been reported to play a critical role in immune response modulation [11,35] (Pshezhetsky and Hinek, 2011; Pshezhetsky and Ashmarina, 2018). Various studies have reported more than one type of immune receptor that is influenced by NEU1 and thus depends on it for its functioning (Pshezhetsky and Hinek, 2011). One such receptor is FcγR, through which NEU1 appears to activate phagocytosis in macrophages [36] (Seyrantepe et al., 2010). Experimental studies have shown that NEU1 expression increases 12 folds when circulating blood monocytes and monocytic cell lines differentiate into macrophages [37] (Liang et al., 2006). Animal studies showed that in a mouse model of 10% NEU1 deficient mice, macrophages and immatures exhibited more sialyation of the cell surface and a simultaneous reduction in the ability of phagocytosis of all types. Importantly, NEU1 deficiency caused this effect *via* a decline in the transduction of signals from the Fc receptors for immunoglobulin G (FcγR) as more sialylation and impaired phosphorylation of FcγR in NEU1 deficient macrophages were noted (Liang et al., 2006; Seyrantepe et al., 2010). Moreover, there is evidence showing sialidase to be overexpressed during the activation of T cells, B cells, macrophages, and neutrophils on the surface of activated T cells, which consequently influences immune function (Amith et al., 2009). Also, it has been demonstrated that endogenous sialidase activity increases considerably during the activation of various immune cells, including T cells, B cells, and monocytes, whereas sialylation of some surface molecules decreases (Landolfi et al., 1985; Landolfi and Cook, 1986). Another receptor important in immunomodulation that has been influenced by NEU1 is the activation of cell surface Toll-like receptors (TLR), which are critical in activating immune responses during infection (Amith et al., 2009). Studies report that ligand-induced activation of TLR-2, -3, and -4 is controlled by NEU1 sialidase activation. Additionally, the interaction of activated NEU1 with TLRs has been reported to promote intracellular signaling as studies show that TLR-4-derived signaling is impaired in the cells of NEU1-deficient mice. Also, the presence of NEU1 is critical for the lipopolysaccharide (LPS)-induced interaction of TLR-4 with the signal transducer protein, MyD88, and activation of the NFkappaB signaling pathway in macrophage and dendritic cell lines (Abdulkhalek et al., 2012; Karmakar et al., 2019). Furthermore, the hyaluronic acid (HA) receptor, CD44, is involved in multiple cell-cell and cell-matrix interactions. Another immune receptor that has been suggested to be influenced by NEU1-mediated desialylation is supported by direct and indirect evidence (Pshezhetsky and Hinek, 2011).

### 4.1 NEU1’s Deficiency Related Immune System Changes and Pathological Menefistaions of Sialidosis

Various studies report a history of recurrent infections in sialidosis patients, attributed to the impact of NEU1 deficiency on the immune system (Monti et al., 2010; Hanamsagar et al., 2012; Li et al., 2021).

### 4.2 NEU1’s Deficiency Related Immune System Changes and Neurodegeneration

As noted earlier, NEU1 plays a critical role in activating cell surface Toll-like receptors (TLR), which are essential in triggering immune responses during infection (Landolfi and Cook, 1986). These TLRs are also present in the central nervous system cells, such as microglia and astrocytes, which are the primary cells responsible for innate immunity in the CNS (Hanamsagar et al., 2012; Li et al., 2021). The expression of TLRs in CNS cells is up-regulated by infection, inflammation, or TLR stimulation, which accelerates the innate immune response. Significantly enough, these TLRs are also known to play a role in various neurogenerative diseases, including AD pathology (Okun et al., 2009; Hanamsagar et al., 2012). Numerous mechanisms have been suggested through which TLR plays a role in AD pathology.

Results of *in vivo* studies in a double transgenic (APPswe/PSEN1dE9) mouse model of AD demonstrated a lack of TLR4 resulted in an increased cortical and hippocampal Aβ load, suggestive of a decisive role of TLR4 in the Aβ clearance by microglial cells (Arroyo et al., 2011).

Another animal study showed TLR2 deficiency was associated with an acceleration of spatial and contextual memory impairment, which was associated with an increase in Aβ42 and transforming growth factor beta1 (TGFβ1) in the brain (Schmidt et al., 1997). Moreover, an accumulating amount of data establishes the link between impaired TLR activation and a subsequent increase in amyloid burden. As noted earlier, as NEU1 is one of the crucial factors in activating the TLR, it is conceivable that NEU1 deficiency may lead to impaired TLR activation (Karmakar et al., 2019), which in turn contributes to AD pathology (Schmidt et al., 1997; Arroyo et al., 2011; Song et al., 2011). In addition, the role of NEU1 in activating macrophages is yet another potential route through which it can be linked to AD pathology (Zhang and Jiang, 2015). Finally, there is convincing evidence that NEU1 is essential for regulating numerous immune activities in the central nervous system (Khan et al., 2021).

Researchers have demonstrated that macrophages isolated from Neu1-deficient mice exhibited a reduction in phagocytosis. Also, the macrophages taken from the Neu1-deficient mice exhibited increased sialylation and impaired phosphorylation of FcR (FcRgamma 1/CD64) and considerably reduced phosphorylation of Syk kinase when treated with IgG-opsonized beads. It is evident that Neu1 activates phagocytosis in macrophages and dendritic cells through desialylation of surface receptors, mainly *via* CD64. Therefore, it can be said that Neu1 is vital for their functional integrity (Liang et al., 2006; Seyrantepe et al., 2010). Moreover, as cells were treated with exogenous Neu1, the phagocytic capability of macrophages appeared to be restored. Hence, it is plausible that FcRg1/CD64 receptors are a substrate of NEU1, and NEU1 activation phagocytosis *via* CD64 receptors may result in an anti-inflammatory environment since this brings microglia/macrophages to the M2 state, which has been reported to be essential in reducing the pathogenesis of neurodegeneration (Sackmann et al., 2017). It is a well-established fact that one of the key issues in AD pathology is the loss of balance of Aβ production and its removal (Selkoe, 1989; Selkoe and Hardy, 2016). Also, there is an elevation in soluble Aβ at an earlier stage, leading to neuronal loss and cognitive impairment and causing abnormal tau phosphorylation, thus perpetrating tauopathy and consequently causing plaque formation (Wisniewski et al., 1990; Fiala et al., 2007). Hence, it is crucial to control the trafficking of soluble oligomers to reduce pathogenesis (Ledo et al., 2013). Studies also show that microglia, instead of performing phagocytic activity, may contribute to pathology (Eikelenboom and Veerhuis, 1996; Eikelenboom et al., 2000; Bennasroune et al., 2019). But stimulating microglia to the M2 phenotype has been shown to increase their anti-inflammatory action. These microglia possess specific markers, notably CD64 (Schmidt et al., 1997; Song et al., 2011). Since NEU1 can potentially activate macrophages into the M2 state, the notion that NEU1 may play a therapeutic role in AD *via* immune activation and immunomodulation seems plausible (Khan et al., 2021).

## 5 NEU1’s Role in Elastin Metabolism

Another critical yet relatively less highlighted role of NEU1 is constituted by the integration of elastin fibers, which is crucial for the integrity of the cardiovascular and respiratory systems and central nervous systems (Bennasroune et al., 2019). NEU1 and its activating partner CathA have been demonstrated to be part of the elastin receptor. Recent studies have highlighted the crucial role in regulating elastic fiber synthesis at various stages. NEU1 plays a critical role in their modulation (Starcher et al., 2008; Antonicelli et al., 2009; Bennasroune et al., 2019). Elastin fibers constitute the major components of the extracellular matrix. They are present abundantly in tissues such as skin, the lungs, and arteries that deal with a high degree of mechanical constraints. In such tissues, elastin is the core component surrounded by microfibril mantles (Antonicelli et al., 2009). The synthesis process of elastic fibers or elastogenesis involves the activity of NEU1, which has been demonstrated by multiple studies (Starcher et al., 2008; Bennasroune et al., 2019). Elastin metabolism is enormously disrupted due to neuraminidase-1 deficiency. In one study of the murine knockout of the Neu-1 gene, an abnormal organization of elastic fibers in the aorta with a reduced level of elastin was noticed. Also, experimental studies on cultured dermal fibroblasts from patients with lysosomal β-galactosidase, PPCA, and Neu-1 deficiencies (such as congenital sialidosis and galactosialidosis (Dickson et al., 1993).

### 5.1 NEU1’s Deficiency-Related Impact on Elastin Metabolism and Pathological Manifestations in Sialidosis

In sialidosis, NEU1 deficiency and its related errors in elastin metabolism manifest typically with abnormalities that are characteristic of the early onset of sialidosis in children (such as failure to thrive, kyphosis, and facial dysmorphism) (Annunziata et al., 2013; Sergi, 2020). This was demonstrated in experiments in which it was noted that elastogenesis in cultured dermal fibroblasts extracted from patients with deficiencies in lysosomal β-galactosidase, PPCA, and Neu-1 deficiencies (including congenital sialidosis and GS) could be reversed by transformation with Neu-1 cDNA, treatment with bacterial sialidase (Wu et al., 2010) or substitutions in the *Neu1*gene (Bonten et al., 2014). These findings suggest a critical role of NEU1 activity in the process of correct deposition and assembly of elastic fibers (Antonicelli et al., 2009; Bennasroune et al., 2019).

### 5.2 NEU1’s Deficiency-Related Impact on Elastin Metabolism and Neurodegeneration

Another important consideration is the role of NEU1 in elastin degradation. Elastin undergoes proteolytic degradation in some physiological and pathophysiological conditions because of a profoundly low turnover rate. Thus, its degradation becomes irrevocable and lasting. This phenomenon leads to the formation of elastin-derived peptides (EDPs) (Ma et al., 2019). A recent growing body of evidence shows that EDP is crucial in the development of numerous age-related vascular diseases. It is known that EDPs can be found in the cerebrospinal fluid of healthy individuals. However, they have been associated with various central nervous system pathologies. For example, their amount has been reported to increase in patients after ischemic stroke (Ma et al., 2019; Ma et al., 2020). Moreover, there is evidence of EDP interfering in the inflammatory process of normal astrocytes. Also, they have been known to cause an increase in the proliferation and invasiveness of astrocytoma and gliomas, which may result in a poor prognosis of central nervous system-related neoplasms. Interestingly, NEU1 has been found to play a vital role in signal processes and biological activities controlled by EDPS. Signaling events typically consist of the phosphoinositide-3-kinase γ (PI3Kγ) pathway and converge to ERK1/2 and Akt activation. This role of EDPs has been linked with cardiovascular disease, respiratory disease, cancer progression, and neurodegeneration. It is to be noted that EDPS has been demonstrated to be involved in the overproduction of beta-amyloid in a model of AD (Ma et al., 2019; Ma et al., 2020; Szychowski et al., 2021), although the precise underlying pathological mechanisms remain unknown.

## 6 NEU1 and Other Cellular Mechanisms

There are various other cellular mechanisms in which NEU1 has been demonstrated to have a critical role. For instance, it is involved in insulin signaling regulation. Multiple studies have elucidated that as insulin attaches to its receptor, the receptor, in turn, rapidly interacts with NEU1 (Dridi et al., 2013). This leads to hydrolyzation of sialic acid residues present in the glycan chains of the receptor with its subsequent activation. Moreover, studies with animal models have shown a decisive role of NEU1 in glucose metabolism and energy signaling. Furthermore, studies of sialidosis patients have shown that the genetic deficiency of NEU1 results in impaired insulin-induced phosphorylation of downstream protein kinase AKT. Notably, treatment with purified NEU1 appeared to restore this impaired signaling. Thus, it is conceivable that NEU1 is important for energy metabolism and the insulin signaling pathway (Dridi et al., 2013; Alghamdi et al., 2014; Fougerat et al., 2018).

Furthermore, various studies indicate the role of NEU1 in acting as a negative regulator of malignant properties of different types of cancer cells (Pshezhetsky and Hinek, 2011). Also, some studies have elaborated on NEU1’s role in regulating the cellular mitogenic response to growth factors. Experiments performed on fibroblasts taken from sialidosis patients exhibited a fairly mitogenic solid response to the same doses of PDGF-BB and IGF-II as compared to fibroblasts of normal skin, indicative of the fact that NEU1 deficiency is associated with a greater number of cell surface receptors remaining sialylated and, consequently, more responsiveness to their respective growth factors (Pshezhetsky and Hinek, 2011).

## 7 NEU1 and Sialidosis

As mentioned before, NEU1 deficiency is most commonly associated with sialidosis (Bonten et al., 1996; Sergi et al., 2001; Sergi, 2020). More than 40 *NEU1* disease-causing mutations have been reported thus far, resulting in sialidosis with varying severity of symptoms (d’Azzo et al., 2015; Khan and Sergi, 2018). The most common mutation to be reported is a missense mutation (Khan and Sergi, 2018). Moreover, since sialidosis is a disease with a diversified range of symptoms with varying degrees of severity, it has been noted that this diversity is essentially due to the degree of activity in the mutant enzyme. This has been attributed to the degree of activity of the mutant enzyme, giving rise to different kinds of NEU1 variants. NEU1 variants have been divided into three categories based on biochemical properties, resulting in different subtypes of sialidosis. The mutant enzyme remains catalytically inactive in the first category and fails to localize to lysosomes. The second category is the one when the mutant enzyme remains inactive; however, it is localized to lysosomes. Finally, the third category is when the mutant enzyme shows residual activity and localizes to lysosomes (Bonten et al., 2000; Khan and Sergi, 2018). Sialidosis is generally divided into two types (Sergi, 2020). Also known as “cherry-red spot myoclonus,” type I sialidosis has been known to be an attenuated and non-neuropathic form of the disease. Late-onset symptoms characterize this type, generally having decreased visual acuity, progressive visual loss, bilateral macular cherry-red spots, gait abnormalities, and myoclonus (Sobral et al., 2014). There are no physical deformities, nor is there any impairment in intelligence. However, myoclonus is often present in type I sialidosis and is the hallmark of this disease (Thomas et al., 1979; d’Azzo et al., 2015). It may initially cause difficulties in fine motor movements and intention tremors but may progress to generalized seizures, which become debilitating with the course of the disease. Thus, despite having normal muscle strength and having average intelligence, patients may become wheelchair users (Caciotti et al., 2020). The degree of severity of the symptoms in patients directly correlates with the type of NEU1 mutations and the amount of residual enzyme activity (d’Azzo et al., 2015). Recent studies have reported that atypical cases of type I sialidosis exhibited myoclonus without visual symptoms and no measurable NEU1 activity. This finding is crucial as it indicates that NEU1 mutations affecting its activity may exist in the absence of characteristic features of sialidosis (Chen et al., 2006; Canafoglia et al., 2014; d’Azzo et al., 2015; Muona et al., 2015; Mohammad et al., 2018; Bou Ghannam et al., 2019). Thus, it is imperative to gather more data regarding the symptoms of atypical cases of sialidosis. Also, it would be worth inquiring about the changes in behavior or other psychological changes in such patients. As mentioned above, decreased activity of NEU1 can cause emotional and behavioral changes in animal models (Ikeda et al., 2021). On the other hand, type II sialidosis is generally regarded as a severe type of disease. Type II sialidosis has been divided into three subtypes: congenital, hydropic, and post-congenital which are characterized by onset *in utero*. This is the most severe kind of disease that may present as hydrops fetalis, neonatal ascites, or both; patients can be stillborn or die shortly after birth following a systemic and fulminant course. The clinical presentation may include facial edema, hepatosplenomegaly, and inguinal hernias at birth. Sialidosis II is an infantile type characterized by the onset between birth and 12 months. Finally, juvenile type has been described by the onset past 2 years of age (Winter et al., 1980; Caciotti et al., 2009; Mitsiakos et al., 2019). The other clinical characteristics of type II include a coarse face, hepatosplenomegaly, dysostosis multiplex, vertebral deformities, and severe mental retardation. The appearance of macular cherry-red spots, myoclonus, hearing loss, and angiokeratoma can be observed in patients who survive for longer. Life expectancy is generally short, although it may vary according to the associated mutations and the intensity of symptoms (Donati et al., 2003; d’Azzo et al., 2015; Caciotti et al., 2020).

### 7.1 NEU1 Deficiency in Sialidosis and Brain Changes

The involvement of the central nervous system in sialidosis is a well-established fact. However, due to the rarity of the disease, most of our understanding of CNS involvement in sialidosis comes from animal model studies (Pshezhetsky and Ashmarina, 2018). Nevertheless, with the help of neuroimaging studies, various changes in sialidosis patients are being documented now (Pshezhetsky and Ashmarina, 2018). Sialidosis II takes a fulminant course, and early demise is the typical fate (Winter et al., 1980; Caciotti et al., 2009). Along with an early-age onset of facial dysmorphism dysplasia, there are neurodegenerative changes in the brain. Neuroimaging studies in sialidosis II patients are limited. However, cerebral ultrasound studies demonstrated pachygyria in the type II sialidosis brain. At the same time, magnetic resonance imaging (MRI) showed corpus callosum hypoplasia with the continual growth of cerebral parenchyma and the dilatation of the occipital horns of the lateral ventricles (Mitsiakos et al., 2019). Hydrocephalus has also been reported in type II sialidosis patients (Donati et al., 2003).

On the other hand, type I sialidosis has a late-onset with central nervous system involvement, typically manifesting as seizures, ataxia, and visual impairment. Therefore, most neuroimaging studies are performed on sialidosis I patients (Pshezhetsky and Ashmarina, 2018). At the onset of the disease, brain MRI may appear normal. However, diffuse atrophy is commonly reported in patients with advanced type I sialidosis. Recent MRI studies of many sialidosis patients have repeatedly reported brain atrophy of varying degrees (Palmeri et al., 2000; Sekijima et al., 2013). MRI studies also show the diffused cortical atrophy compromised white matter integrity, more pronounced in the occipital lobe. Additionally, decreased connectivity from the temporal and occipital lobes to the hippocampus and para-hippocampus has also been noted. Moreover, a compromised posterior visual pathway, with extensive involvement of the brain’s posterior part, has been related to cortical blindness in sialidosis I patients (Lai et al., 2009; Sobral et al., 2014; Lu et al., 2017; Gultekin et al., 2018; Hu et al., 2018; Coppola et al., 2020).

## 8 NEU1 as a Therapeutic Target, Therapeutic Interventions for Sialidosis: The Obstacles and the Progress So Far

The rarity of sialidosis and the unique biochemical nature of NEU1 are the main hindrances to developing an efficacious sialidosis treatment. However, enzyme replacement therapy (ERT) with recombinant lysosomal hydrolases has been successfully used for various non-neuropathic LSDs (Wang et al., 2005). However, it has not been effective in sialidosis, attributable to the unique properties of NEU1. Firstly, due to the absence of a functional mannose-6-phosphate recognition marker, NEU1 is not endocytosed by mammalian cells. Therefore, researchers used a recombinant NEU1 enzyme in one study, which was purified from overexpressing insect cells, and attempted ERT in Neu1^−/−^ mice. There was a considerable improvement in symptoms in systemic organs after an increased initial level of NEU1 enzyme activity with a subsequent considerable reduction of lysosomal storage. Unfortunately, after 2 weeks of treatment, a severe immune response was observed in the mice towards the exogenous NEU1 enzyme. This immunogenicity of NEU1 makes it challenging to provide enzymatic replacement therapy (Wang et al., 2005). Another feature of NEU1 that makes it challenging to use as therapy is that it tends to aggregate, and its activity depends strictly on its binding with PPCA (d’Azzo et al., 2015). However, it has been suggested that NEU1’s strict dependence of the enzyme on PPCA for catalytic activation may provide its therapeutic benefits (Sobral et al., 2014). Hence, chaperone-mediated therapy has been proposed and tested in mouse models (Bonten et al., 2013). Initially, a new mouse model of the non-neuropathic attenuated type I form of sialidosis was developed, carrying a V54M amino acid substitution, which has been identified in type I sialidosis patients. In these mice, a scAAV-based PPCA-mediated chaperone gene therapy study was conducted. In one-year-old *Neu1* ^*−/−*^; *NEU1* ^*V54M*^ mice with signs of type I sialidosis pathology, a single dose of the recombinant AAV vector was injected, and then these mice were sacrificed a month later. It was observed that such therapy resulted in an overall improvement of tissue pathology. Furthermore, with an increased expression of the PPCA enzyme, in the liver of the injected mice, a 3-fold increase of the NEU1^V54M^ basal activity in all the tested tissues was reported. This pharmacologic, chaperone-mediated therapy has been regarded as a promising approach for other *NEU1* mutations found in patients with type I sialidosis (Bonten et al., 2013). In addition, the possibility of environmental factors affecting the residual enzymatic activity was suggested previously and has been confirmed by more recent studies (d’Azzo and Annunziata, 2020). Researchers performed experiments to find the role of the epigenetic component in the regulation of *NEU1* gene expression. It was observed that inhibiting histone deacetylases (HDACi) leads to an upregulation of NEU1 transcription as well as enzyme levels, consequently increasing its activity. HDACs are known to be involved in the regulation of cellular pathways by causing repression of metabolic gene transcription (d’Azzo and Annunziata, 2020). Thus, the treatment of *NEU1* mRNA expression with HDACi caused an upregulation of the levels of mutant *NEU1* mRNA as well as an increase in the residual activity in fibroblasts extracted from patients with Sialidosis. It is noteworthy here that inhibition of HDACi also led to an increase in the transcription of PPCA, NEU1, and β-galactosidase in the complex. Hence, it is conceivable that epigenetic factors are another way to increase NEU1 activity. Further research in this area is imperative to understand how to target NEU1 to alleviate the symptoms (d’Azzo and Annunziata, 2020). Furthermore, another recent discovery in this vein is the potential role of dietary modification in enhancing the residual NEU1 activity. In a recent study, the effect of recombinant protective protein/cathepsin A (PPCA), along with pharmacological agents and dietary compounds, was examined on the residual activity of mutant NEU1on the primary fibroblasts of a small cohort of patients with sialidosis I (Mosca et al., 2020). The study reported a small yet consistent increment in NEU1 activity in most of the tested fibroblasts. Interestingly, this study reported the beneficial effects of betaine, a natural amino acid derivative, in type I sialidosis, the less attenuated form of the disease. It was observed that betaine was administered in mouse models with residual NEU1 activity, thus mimicking type I sialidosis. An increase was noticed in levels of mutant NEU1, but oligosacchariduria was also observed to be resolved. The notion of dietary supplements providing beneficial results for sialidosis is hopeful. More studies are warranted to confirm these results and see if such dietary modification also brings any improvement in the symptomatology of sialidosis (Mosca et al., 2020). It is worth mentioning here that dietary supplementation of betaine has also been beneficial in other neurodegenerative diseases (Sun et al., 2017; Zhao et al., 2018). All in all, the advancement in the understanding of NEU1 activity being enhanced by environmental factors can be profoundly helpful in establishing a broader range of therapeutic interventions for sialidosis patients. Lastly, there is also evidence of testing the treatment of bone marrow transplant (BMT) in type I sialidosis patients. After BMT, despite some preservation in some neurological areas, an overall decline in motor performance was noticed. Also, hematopoietic cell transplantation has been tried in a patient with type II sialidosis, but the results were inconclusive (Gupta et al., 2022).

## 9 NEU1 and Alzheimer’s Disease. Involvement of More Than One Cellular Mechanisms

In our previous study, we elaborated on the role of NEU1 in sialidosis and its role in AD via the immune system. Since NEU1 deficiency causes impaired phagocytosis, we hypothesized that NEU1 could be a potential therapeutic target for AD as it may enhance effective phagocytosis in the AD brain (Khan et al., 2021). In the current studies, we aimed to highlight and summarize various other cellular mechanisms in which NEU1 has been reported to be involved in recent years. For example, impaired TLR activation is another mechanism that causes an increase in amyloid burden and, thus, causes AD pathology. NEU1 deficiency has been linked with impaired TLR activation. Thus, making the notion plausible that NEU1 is linked with AD pathology *via* an impaired TLR activation [41–44]. However, underlying pathological mechanisms need to be explored further. Additionally, NEU1 being the negative regulator of lysosomal exocytosis has been extensively discussed in the previous literature. The changes noticed in the brains of *Neu1* ^−/−^ mice are similar to those of AD. The over-sialyation of amyloid precursor protein APP has been attributed to a lack of NEU1 deficiency. Moreover, the aggregation of these over-sialylated APP in the lysosomes, with the extracellular release of Aβ peptides *via* excessive lysosomal exocytosis, is attributed to NEU1 deficiency. As the cerebral injection of NEU1 caused a decline in β-amyloid plaques, it is possible that NEU1 could be a potential therapeutic target for the AD brain.

Importantly, similar changes were also noticed in the Zebrafish model as well. Furthermore, in the brain of Neu1-KO zebrafish, an altered pattern of aggregation of GM1 ganglioside was noted (Ikeda et al., 2021). This finding establishes yet another link between NEU1 and AD pathology. As mentioned before, GM1 ganglioside has been demonstrated to play a critical role in AB pathology by causing an increase in AB production. Hence, the accumulation of GM1 in a NEU1 deficient brain, which has exhibited changes similar to AD, further strengthens the notion that NEU1 deficiency may contribute to AD pathology. Hence, it is plausible that NEU1 scarcity may lead to ganglioside accumulation which contributes to pathological changes related to AD (Ikeda et al., 2021). The role of elastin derived peptides (EDP) and AD pathology hints toward yet another link between AD pathology and NEU1. As noted earlier, EDPs are the products of elastin degradation and have been associated with numerous central nervous system pathologies. Notably, it seems that EDPs are also involved in the overproduction of beta-amyloid, although the underlying mechanisms remain unclear. Interestingly, some research has elucidated that NEU1 plays an essential role in numerous biological processes controlled by EDP. Moreover, NEU1 is also involved in the signaling process related to EDPs. Signaling events involving NEU1 consist of a phosphoinositide-3-kinase γ (PI3Kγ) pathway, with convergence to ERK1/2 and Akt activation. Further research in this area may provide more details on this crucial link.

Another mechanism that potentially links NEU1 to AD is *via* G protein-coupled receptor (GPCR) kinases (GRKs) (Tembely et al., 2022). G protein-coupled receptor (GPCR) kinases (GRKs) are essentially a family involving seven serine/threonine kinases (GRKs 1–7) that are involved in the phosphorylation and desensitization of GPCRs. However, evidence shows that GRKs are also associated with phosphorylation of non-GPCR proteins to manipulate various other cellular responses besides GPCR-dependent mechanisms. GRKs have also been reported to be involved in the pathological phosphorylation and accumulation of tau and amyloid pathology in AD brains (Guimaraes et al., 2021). Studies have suggested their unique role in the pathological processes involved in AD, and thus, they can be a potential therapeutic target. Studies have also indicated that the pattern of expression of the GRKs in neurons is cell type-specific in the human brain in AD subjects. Additionally, an overall positive correlation has been established between GRKs 2, 3, and 6 and soluble tau found in the AD brain (Guimaraes et al., 2021). Furthermore, it has been suggested that these kinases may have direct or indirect involvement in the altered tau solubility, tau phosphorylation, as well as tau aggregation. Interestingly, there is a line of evidence that elaborates cross-talk between GPCR and the matrix metalloproteinase 9 (MMP-9) and Tyrosine kinase receptors (RTK) or TLRs signaling pathways where NEU1 plays a critical role (Cattaneo et al., 2014; Tembely et al., 2022). This provides a unique role for NEU1 in receptor transactivation processes. Researchers have found that as GPCR agonists bind to their respective cognate receptors, induction of GPCR-signaling processes using the Gα_i_ proteins, as well as MMP-9 activation, takes place, which results in an elevation of NEU1 sialidase activity. As a result of this, the increased sialidase activity of NEU1, which is bound to the RTK or TLR, leads to hydrolyzation of α-2,3-sialyl residues of the receptor, ultimately causing an RTK or TLR activation. Thus, another cellular mechanism can be noted, connecting NEU1 to AD pathology, making it a potential therapeutic target.

## 10 Further Perspective and Conclusion

An enormous amount of research has been done in recent years exploring the role of *Neu1* in numerous cellular mechanisms, and more substrates of NEU1 are being reported over time. Interestingly, some researchers have also found a role for NEU1 in the infection caused by the SARS-CoV-2 virus (Bongiovanni et al., 2021). From the discussion in this paper, it is clear that NEU1 plays a key role in a variety of cellular mechanisms, many of which are directly or indirectly associated with neurogenerative pathologies in rare diseases like sialidosis and also have been linked to highly prevalent neurodegenerative diseases like AD. We hypothesize that in the near future, there will be some evidence of a potential contributory role of NEU1 in various non-neurologic degenerative diseases. Some studies have suggested that inhibiting Neu1 activity may positively affect drug-induced liver injury (Chen et al., 2020). Down-regulation of NEU1-regulated pathways seems to reduce the progression of various types of cancer (O’Shea et al., 2014; Machado et al., 2015). Importantly, a few studies report that inhibition of surface or secreted neuraminidase may benefit chronic neuroinflammation and subsequent microglia-mediated neurodegeneration (Allendorf, 2021). On the other hand, studies report that elevating the NEU1 levels seems to have a positive, beneficial impact on sialidosis-related CNS neurodegeneration (d’Azzo et al., 2015; Khan and Sergi, 2018). Substantially, all these investigations point to the profoundly crucial role of NEU1 in cytologic degeneration, making it a unique and important therapeutic target for neurodegeneration. We hope that these and other studies may trigger more robust investigations to pinpoint the role of NEU1 in cellular mechanisms associated with neurobiology and neurophysiology. It is our intention to help identify therapeutic strategies for diseases like AD, which continue to indelibly associate the elderly in the 21st century.
